# Color change of tooth-colored restorative materials bonded to silver diamine fluoride-treated dentine: a systematic review

**DOI:** 10.1186/s12903-024-04487-0

**Published:** 2024-06-27

**Authors:** Sedigheh Sabbagh, Taraneh Movahhed, Hossein Bagheri, Masoumeh Sadeghi, Saroash Shahid, Homeira Mohammadi

**Affiliations:** 1 Dental Materials Research Center, Mashhad University of Medical Sciences, Mashhad, Iran; 2 Department of Pediatric Dentistry, Faculty of Dentistry, Mashhad University of Medical Sciences, Vakilabad Blvd, Mashhad, Iran; 3 Department of Epidemiology, School of Health, Mashhad University of Medical Sciences, Mashhad, Iran; 4 Dental Physical Sciences Unit, Centre for Oral Bioengineering, Institute of Dentistry, Queen Mary University of London, London, UK; 5 Metabolic Syndrome Research Center, Mashhad University of Medical Sciences, Mashhad, Iran

**Keywords:** Color, Composite resins, Dental caries, Glass ionomer cements, Silver diamine fluoride, Systematic review

## Abstract

**Background:**

The desirable properties of silver diamine fluoride (SDF) make it an effective agent for managing dental caries and tooth hypersensitivity. There are several clinical instances that SDF application might precede the placement of direct tooth-colored restorations. On the other hand, SDF stains demineralized/carious dental tissues black, which might affect the esthetic outcomes of such restorations. Color is a key parameter of esthetics in dentistry. Therefore, this study aims to systematically review dental literature on color/color change of tooth-colored restorations placed following the application of SDF on dentine.

**Methods:**

Comprehensive search of PubMed, Embase, Scopus and ISI Web of Science databases (until August 2023) as well as reference lists of retrieved studies was performed. In vitro studies reported color or color change of tooth-colored restorative materials applied on SDF-treated dentine were included. Methodological quality assessment was performed using RoBDEMAT tool. Pooled weighted mean difference (WMD) and 95% confidence interval (95% CI) was calculated.

**Results:**

Eleven studies/reports with a total of 394 tooth-colored restorations placed following a) no SDF (control) or b) SDF with/without potassium iodide (KI)/glutathione dentine pre-treatments were included. Color change was quantified using ∆E formulas in most reports. The pooled findings for the comparison of resin-based composite (RBC) restorations with and without prior 38% SDF + KI application revealed no statistically significant differences in ∆E values at short- and long-term evaluations (~ 14 days: WMD: -0.56, 95% CI: -2.09 to 0.96; I^2^: 89.6%, and ~ 60 days: WMD: 0.11; 95% CI: -1.51 to 1.72; I^2^: 76.9%). No studies provided sufficient information for all the items in the risk of bias tool (moderate to low quality).

**Conclusions:**

The limited evidence suggested comparable color changes of RBC restorations with and without 38% SDF + KI pre-treatment up to 60 days. The included studies lacked uniformity in methodology and reported outcomes. Further studies are imperative to draw more definite conclusions.

**Protocol registration:**

The protocol of this systematic review was registered in PROSPERO database under number CRD42023485083.

**Supplementary Information:**

The online version contains supplementary material available at 10.1186/s12903-024-04487-0.

## Background

Untreated dental caries is a major public health issue, affecting more than one third of the population worldwide [1]. In recent years, the emphasis in delivering dental care has been shifted towards preventive and minimal intervention dentistry (MID) approaches [2]. The MID approach generally focuses on the interception of oral diseases at an early stage. In caries management, MID not only can result in preserving tooth structure and ultimately extending tooth lifespan, but is a potential cost-effective solution for tackling the global burden of untreated caries. The use of silver diamine fluoride (SDF) is one of the MID protocols relevant to caries management [3], and its effectiveness in arresting dental caries in children and adults has been supported by systematic reviews [4–6]. 

SDF was first developed in the 1960s in Japan, and about half a century later, in 2014, was cleared by the United States Food and Drug Administration as an anti-hypersensitivity agent [7]. This colorless alkaline solution is generally used at 38% concentration for managing dental caries and treating dentine hypersensitivity. SDF exerts its effects by disrupting biofilm formation, enhancing remineralization, counteracting demineralization, occluding dentinal tubules, and preventing collagen degradation [8]. There are several clinical instances that SDF application might precede direct restoration placement both in children and adults [9]. One example is using SDF for general caries control in high-risk patients with or without behavioral/medical conditions until performing conventional restorative treatments is feasible [9–11]. Moreover, SDF can be used alongside atraumatic restorative treatment to manage dental caries as a same-day or multiple-appointment silver-modified atraumatic restorative treatment [11–13]. Other examples include using SDF as an indirect pulp capping material [14, 15], and for managing dentine hypersensitivity or symptoms of molar-incisor hypomineralisation-affected teeth prior to restorative procedures [9]. In addition, SDF-treated teeth might be restored for enhancing esthetics [8, 16], and/or restoring form and function [11].

SDF as a simple, effective, and efficient treatment is safe with no acute systemic complications [8]. Nonetheless, its main disadvantage is black discoloration of carious lesions attributed to the formation of silver compounds on carious tooth surface, comprising esthetics [8, 17]. This black staining could be a barrier to SDF widespread use [18]. It has been suggested that developing a way to minimise such staining would increase SDF acceptability [12, 18]. Several approaches have been proposed to overcome this significant drawback, however, no definite clinical solution is currently available [17]. Potassium iodide (KI) solution applied following SDF resulted in a significant reduction in black staining in several studies [19]. However, its long-term effectiveness has not yet been proven [19, 20]. Glutathione (GSH) mixed with SDF have been evaluated in few studies showing positive results, although not superior to those of KI [21, 22]. Furthermore, new formulations based on silver nanoparticles causing no obvious staining are considered promising alternatives to the existing SDF formulations. These new formulations are still under investigation and no commercial products are available for their widespread use [23].

Improvements in dental materials/techniques, and patients’ demand for esthetic restorations have contributed to the recent significant increase in request for tooth-colored restorations [24]. Color is a key parameter of esthetics in dentistry [25], evaluated by visual judgment or measuring instruments [25, 26]. Despite initial promising outcomes, the color of esthetic restorations might change over time, affecting their clinical longevity [27]. On the other hand, considering the increased interest in use of SDF in recent years [7], its application before tooth-colored restorations possibly accelerates undesirable color changes [7, 15, 28, 29]. Therefore, investigating (alterations in) the color of direct restorative materials bonded to SDF-treated teeth is timely and worthy of attention.

The primary aim of the present study is to systematically review and summarize current laboratory evidence reported color/color change of tooth-colored restorations placed after SDF application on dentine. We also addressed the following issues when reported in the included studies (secondary outcomes): 1) if color changes were clinically perceptible/acceptable, and 2) presence/amounts of marginal discoloration.

## Methods

This systematic review adhered to the Preferred Reporting Items for Systematic Reviews and Meta-analyses (PRISMA) 2020 Statement [30]. The protocol of this study was registered in the PROSPERO (International Prospective Register of Systematic Reviews) database (CRD42023485083).

### Eligibility criteria

The inclusion criteria were: (1) studies evaluating optical properties of tooth-colored restorations placed after the application of SDF solutions (any modifications to SDF application protocol, including the use of KI or GSH, were acceptable); (2) study outcome must be color and/or color changes of the restorations; (3) interventions must be applied on natural (human or animal) dentine; (4) in vitro studies. When applicable, data of negative controls (restorations with no previous/only water dentine pre-treatments) were also collected. The exclusion criteria were: (1) studies/groups in which the samples were not restored following SDF application; (2) evaluating other formulations containing silver (including nano silver fluoride and silver nitrate) or other remineralizing agents; (3) other study designs.

Data pooling was performed for comparisons with similar dentine pre-treatments, restorative materials, outcome measures, and assessment time points.

### Information sources and search strategy

Two reviewers (SeSa and TM) independently developed search strategy and discrepancies were resolved by consensus. Four electronic databases (PubMed, Embase, Scopus and ISI Web of Science) were systematically searched until August 2023, with no restriction on language and publication dates. The following keywords were used: ("silver diamine fluoride" OR "silver diammine fluoride" OR "silver ammonia fluoride" OR "diamine silver fluoride" OR "diammine silver fluoride" OR "silver fluoride" OR "SDF") AND (masking OR mask OR masked OR color OR colour OR discoloration OR discolored OR discolouration OR discoloured OR staining OR stained OR stain OR esthetic OR aesthetic OR visual OR restoration OR restorative OR restored OR filling OR filled). Furthermore, reference lists of included studies and relevant systematic reviews [17, 19, 31] were manually searched for additional pertinent studies. The search strategy is presented in Supplementary material 1.

### Selection process and data extraction

Two independent reviewers (SeSa and HM) screened titles and abstracts of records retrieved from electronic search to find eligible reports, using EndNote software (version X9, Clarivate Analytics, Philadelphia, PA, USA). This was followed by examining the full texts of selected records to confirm that they met the inclusion criteria. Any disagreements were resolved by discussion.

The same two reviewers independently performed data extraction. The main characteristics of the included studies were as follow: author’s name, publication year, country, tooth type, tooth and dentine caries status, sample size and preparation methods, materials used and their application methods/times, aging/storage methods/solutions, outcome measures, assessment times and methods, and main findings. More information on missing/unclear data were collected by contacting corresponding authors. Any disagreements were resolved by discussion or further consultant with a third reviewer (HB).

### Study risk of bias assessment

Two independent reviewers (SeSa and HM) assessed the risk of bias of included studies based on RoBDEMAT tool. This tool contains four domains and a total of nine items: bias related to planning and allocation (control group, randomization of samples, and sample size rationale and reporting), specimen preparation (standardization of samples and materials, and identical experimental conditions), outcome assessment (adequate and standardized testing procedures and outcomes, and blinding of the test operator), and data treatment and outcome reporting (statistical analysis, and reporting study outcomes). Each item was judged as either “sufficiently reported/adequate”, “insufficiently reported”, “not reported/not adequate” or “not applicable”. No summary score was generated based on recommendation [32]. Any disagreements in methodological quality assessment were resolved by discussion or further consultant with a third reviewer (MS).

### Data pooling and synthesis

The color difference (∆E), and the amount or changes in CIEL*a*b* color coordinates (L*, a*, and b*) were considered as outcome measures for pooling the data. When required, the duration of aging procedures was estimated based on days according to the available data. Pooled weighted mean difference (WMD) and 95% confidence interval (95% CI) was calculated using Stata software (version 17.0, StataCorp, Collage Station, Texas, USA). Meta-analysis was generally not performed due to substantial setting/methodological and conceptual heterogeneity in the comparison groups and outcomes, and also small number of studies in the considered comparisons.

## Results

### Study selection

**Fig. 1:**
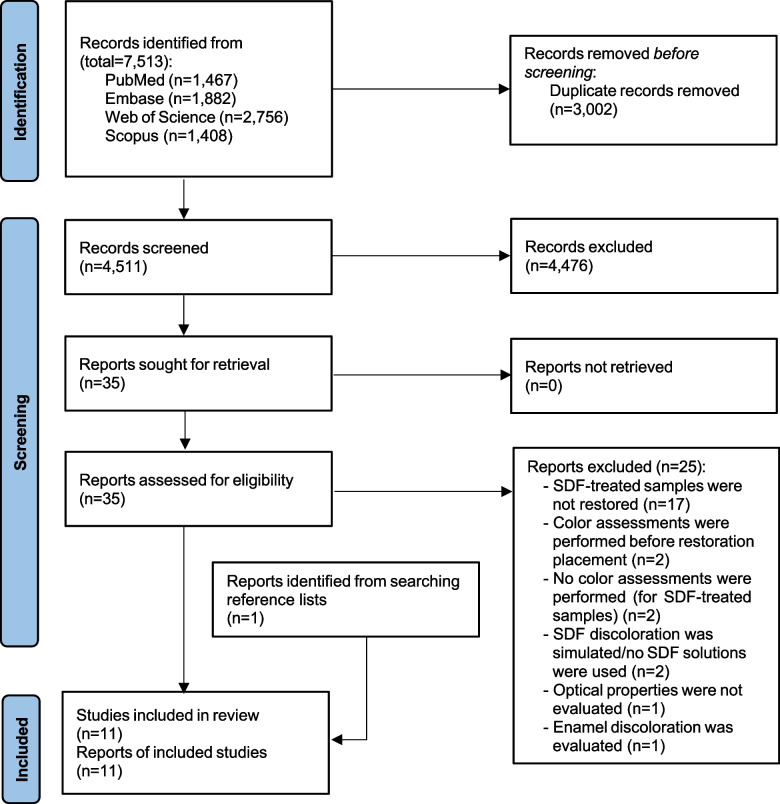
Flow diagram of the study selection. SDF: Silver diamine fluoride

A total of 7,513 records was identified through electronic search. After removing duplicates and screening the titles and abstracts of the remaining 4,511 records, 35 reports were selected for full-text assessment. Of those, 25 reports did not meet the inclusion criteria mainly due to not restoring SDF-treated samples and, therefore, were excluded from the systematic review (Supplementary material 2) [16, 22, 23, 29, 33–53]. Finally, 10 studies/reports from electronic search [7, 21, 28, 54–60], and one study/report [61] retrieved through handsearching were included in the systematic review (Fig. 1). In the six included studies, those groups in which samples were not restored [7, 54, 56, 61], interventions were applied on enamel [58, 61], and/or other remineralizing agent was used [55] were further excluded. Besides, outcomes from two studies judged to have no significant setting/methodological heterogeneity were pooled [7, 55].

### Study characteristics

Table 1 presents the summary of study characteristics. All included studies were published between 2016–2022, with most (nine studies) published during 2021–2022. Authors from Saudi Arabia, with three articles, published the highest number of reports [28, 54, 58], followed by those from Brazil [7, 55], India [21, 59], and The United States of America [57, 61] each with two published articles. In addition, one study was a scientific collaboration between Germany and Egypt [56]. Masking ability [56, 60] and color stability [28, 55] each were evaluated by two studies, while the rest of the reports aimed to assess staining/discoloration/darkening/color change [7, 21, 54, 57–59, 61]. Although the total number of included samples from each eligible report was varied from 10 to 90 (total: 394, mean: 35.8 per study), the number of samples in each included group/subgroup was ranged from 1 to 18. Except two [7, 55] and one [57] studies using bovine teeth/incisors and did not mentioned the tooth type, respectively, all other reports included human teeth (mostly posterior). Only one study considered the color similarity of collected/included teeth [55]. Several distinct approaches were used for storage and/or disinfection/sterilization of the collected extracted teeth. However, disinfection/sterilization method was not specified in five studies [7, 54–56, 60]. Tooth substrates were natural carious dentine [21, 54, 56, 57, 59], artificially demineralized dentine [7, 28, 55], or sound dentine [28, 58, 60].

**Table 1:** Main characteristics of included studies in the systematic review of color change of tooth-colored restorative materials bonded to silver diamine fluoride-treated dentine

No	Author, year of publication, country	Tooth type (total sample size)/caries status of teeth	Storage and preparation of samples	Dentine pre-treatments (for each restorative material)			Restorations			Outcome(s) and color assessment times and method(s)
				**Agent** (#*n*)	**Brand**	**Application method**	**Material**	**Brand**	**Characteristics and process**	
1	Aldosari [28], 2022, Saudi Arabia	Premolars (*n* = 90)/sound (caries-free)	Storage: 0.5% chloramine-T (4–7 °C) Preparation: - Exposing occlusal dentine - Performing dentine demineralization for 7 d (except for control sub-groups)	Control (*n* = 10)	NA	No treatment	RBC	RBC: Neo Spectra ST LV; Dentsply Sirona, Pennsylvania, USA (shade: A1)	Time: immediately/delayed (1 w) Thickness: 2 mm Aging/storage: Storage in distilled water at 37 °C	Outcomes: 1- ΔECIEL*a*b* (ΔE > 3.3 was considered significant) 2- L*, a*, b* Timepoints: 1- 1 d after restoration 2- 7 d after restoration Method: Using a SPM relative to the standard D65 (LabScan XE; Virginia, USA)
				38% SDF (*n* = 10)	SDF: Advantage Arrest; Elevate Oral Care, Florida, USA	SDF: 1 drop was applied and dried				
				38% SDF (delayed restoration) (*n* = 10)						
							RMGI	RMGIC restorative material: Fuji II LC CAPSULE; GCC, Tokyo, Japan (shade: A1)		
							GIC	GIC restorative material: Fuji IX; GCC, Tokyo, Japan (shade: A1)		
2	Alsagob [54], 2022, Saudi Arabia	Posterior permanent teeth (*n* = 40)/carious (cavitated carious lesions with ICDAS score: 5)	Preparation: Teeth were sectioned into two parts	38% SDF (*n* = 10)	SDF: Advantage Arrest, Elevate Oral Care, West Palm Beach, FL, USA	SDF: applied for 1–2 min	RBC	Resin composite: light-cured, Clearfil Majesty, Kuraray, NY, USA (shade A2)	Time: immediately/delayed (2 w) Thickness: 2mm Process: 1- Acid etch 2- Adhesive 3- Filling: using incremental technique, LCT: 40s Aging/Storage: Storage in distilled water	Outcome: - ∆E2000 Timepoints: 1- Baseline (before interventions) 2- After SDF application 3- After restoration (only for immediately applied restorations) 4- After 2 w (only for immediately applied restorations) 5- After 4 w Method: Using LabScan XE SPM (HunterLab, Reston, VA, USA)
				38% SDF (delayed restoration) (*n* = 10)			GIC	GIC restorative material: self-cured, Ketac Fil Plus Aplica, 3M, Maplewood, MN, USA (shade A2)	Time: immediately/delayed (2 w) Thickness: 2mm Process: Filling: using incremental technique Aging/Storage: Storage in distilled water	
3	Ferreira [55], 2022, Brazil	Bovine incisor teeth (*n* = 60, Initial color ranges: L*(87–93), a* (0–1), b* (8–30))/sound (caries-free)	Storage: Distilled water Preparation: - Preparing cavities - Submitting to cariogenic challenge for ~ 14 d - Excavating infected dentine	12% SDF + 10% KI (*n* = 10)	SDF: Cariestop, Biodinâmica, Ipiborã, PR, Brazil KI: Farmácia Liane, Rabeiro Preto, SP, Brazil	1- SDF: 1 drop for 2 min 2- KI: 2 drops/applications for 2 min 3- Rinsing (distilled water) and drying (absorbent paper)	RBC	Composite resin: Filtek Z350, 3 M ESPE Dental Products, St. Paul, MN, USA	Thickness: 2mm Process: 1- Adhesive system (universal): used in self-etch mode, LCT: 10 s 2- Filling: using incremental technique, LCT: 20 s Aging: 1- Storage in artificial saliva at 37 °C for 24 h 2- TMC (~ 5 years, 1,200,000 cycles)	Outcomes: 1- ΔE2000 2- ∆L*, ∆a*, ∆b* Timepoints: 1- After restoration 2- After TMC 3- 30 d after TMC Method: - Using a SPM (EasyShade, VITA Zahnfabrik, BadSckingen, Germany) - Over a white background and inside a standardized lightbox with D65 illuminant
				38% SDF + KI (*n* = 10)	SDF, KI: Riva Star, SDI, Baywater, Victoria, Australia	As PMI 1- SDF: 1 drop for 1 min 2- KI: 2 applications/drops for 2 min 3- Rinsing and drying (absorbent paper)				
							RMGI	RMGIC: Vitremer, 3 M ESPE Dental Products, St. Paul, MN, USA	Thickness: 2mm Process: 1- Primer (LCT: 20 s 2- Filling: using bulk-fill technique, LCT: 40 s 3- Finishing gloss (LCT:20 s) Aging: 1- Storage in artificial saliva at 37 °C for 24 h 2- TMC (~ 5 years, 1,200,000 cycles)	
				Control (*n* = 10)	NA	No treatment				
4	Raafat [60], 2022, Egypt	Human premolar teeth (*n* = 36)/NM	Storage: Distilled water Preparation: - Preparing cavities (sound dentine) - Cavity conditioning with %10 polyacrylic acid	38% SDF + KI (*n* = 6)	SDF, KI: Riva Star, SDI, Bayswater, Australia	As PMI 1- SDF 2- KI: applied until the white reactionary precipitate was formed 3- Washing (water)	ZRGI	ZRGI: Zirconomer Improved, Shofu Inc., Japan	Thickness: 2 mm Process: 1- Filling 2- Finishing and polishing	Outcome: - ΔECIEL*a*b* Timepoints: 1- Baseline (sound dentine) 2- After SDF ± KI application 3- After restoration Method: - Using a reflective SPM (RM200QC, X-Rite, Germany) related to the standard illuminant D65 - Using a white background
				38% SDF (*n* = 6)	SDF: Riva Star, SDI, Bayswater, Australia	SDF: as PMI	RMGI	RMGI restoration: Fuji II LC, GC, Tokyo, Japan		
							GIC	High viscous GI restoration: Fuji IX, GC, Tokyo, Japan		
5	Sakr [58], 2022, Saudi Arabia^a, b^	Human premolars (*n* = 10)/no caries (free of caries, previous restorations or stains)	Storage: 0.1% thymol solution (4 °C) Preparation: Preparing class I cavities (1mm into dentine)	38%SDF + KI (*n* = 10)	SDF: Fagamin, Tedequim KI liquid: Lugol’s sol (distilled water 85% KI 5%), J. Crow Company	1- SDF 2- KI 3- Washing (water) and drying (compressed air)	GIC	Self-cure GIC: Harvard Ionoglas Fill Extra OptiCaps (shade A2)	Process: 1- Conditioner 2- Filling 3- Protective coating: LCT: 20 s Aging: Storage in artificial saliva	Outcomes: 1- ΔE2000 2- ΔL* Timepoints: 1- Immediately after restoration 2- After 1 w 3- After 2 w 4- After 3 w 5- After 4 w Method: Using Nix Pro color sensor (Canada) using industry standard 45/0° measurement
6	Fröhlich [7], 2021, Brazil	Bovine incisors (*n* = 15)/NM (free of cracks and opacities)	Preparation: - Preparing dentine blocks - Submitting to cariogenic challenge by pH cycling for 14 d	Control (*n *= 5)	NA	No treatment	RBC	Composite resin: Filtek Z350 XT A2d, 3M ESPE (dentine shade)	Time: immediately Thickness: 1 mm Process: 1- Acid etch 2- Adhesive system 3- Filling (LCT: 20 s) Aging: Storage in deionized water at 37°C	Outcomes: 1- ΔE2000 2- Clinical acceptance (threshold = ΔE > 1.8) Timepoints: 1- Baseline (after restoration) 2- After 7 d 3- After 14 d 4- After 30 d 5- After 60 d Method: - Using a digital SPM (SP60, X-Rite, Grandville, Mich., USA) in analyzing mode using a D65 illuminant - Coupling medium: glycerin - Using a neutral gray background
				38% SDF (*n* = 5)	SDF: Step 1, Riva Star, SDI, Bayswater, Australia	As PMI SDF: 1 application for 60 s				
				38% SDF + KI (*n* = 5)	SDF: Step 1, Riva Star, SDI, Bayswater, Australia KI: Step 2, Riva Star, SDI	As PMI 1- SDF: 1 application for 60 s 2- KI: applied until the formed creamy white color turned clear (The products were left to dry) 3- Washing (water, at least 10 s) and air-drying				
7	Hamdy [56], 2021, Germany + Egypt	Primary molars (*n* = 26)/carious (occlusal caries extending to dentine)	Storage: Distilled water (room temperature) Preparation: Preparing carious dentine slices	38% SDF (*n* = 13)	SDF: Riva Star, SDI GmbH, Germany	1- SDF: 1 drop for 1 min 2- Rinsing (water, 30 s) and drying (with air)	RBC	Universal composite restorative material: Filtek Z250, 3 M ESPE, USA (shade A1)	Thickness: 4 mm Process: 1- Adhesive (universal self-etching) 2- Filling: using incremental technique, LCT: 20 s Aging: Suntest aging for 24 h in water at 37°C	Outcomes: 1- ΔECIEL*a*b* 2- ΔL/L*, Δa/a*, Δb/b* Timepoints: 1- Baseline (before interventions) 2- After restoration 3- After aging Method: Using a SPM (X-Rite SP62, X-Rite GmbH, Cologne, Germany) adjusted on a D-65 standard illuminant
							GIC	Fast-setting GI restoration: Fuji IX Gp, GC Corporation, Japan (shade A1)	Thickness: 4 mm Process: 1- Dentine conditioner 2- Filling: using bulk-fill technique 3- Topcoat: LCT: 20 s Aging: Suntest aging for 24h in water at 37°C	
8	Kamble [21], 2021, India	Primary molars (*n* = 30)/carious (lesions radiographically involving enamel and dentine)	Storage: - Distilled water - Disinfection: 0.5% NaOCl for 1 w Preparation: - Performing complete caries excavation (cavity walls were caries-free) - Preparing the cavities	38% SDF (*n* = 10)	SDF: Fagamin, Argentina	As PMI 1- SDF: applied and agitated for 1 min, left for 2 min 2- Rinsing (water, for 30 s)	GIC	GIC: GC corporation, Tokyo, Japan	NM	Outcomes: 1- ΔECIEL*a*b* 2- Discoloration/color change (visual examination) Timepoints: 1- Day 1 2- 1 w 3- 4 w Methods: 1- Visual examination: using photographs 2- Color measurement: using a SPM (UV-NIR-3600 Shimadzu, Japan, range: 400–700 nm)
				38% SDF + KI (*n* = 10)	SDF: Fagamin, Argentina Saturated KI solution: Lugol’s solution, India	As PMI 1- SDF 2- KI: reapplied until the creamy white precipitate became clear 3- Rinsing (water, for 30 s)				
				38% SDF + GSH (*n* = 10)	SDF: Fagamin, Argantina GSH: Health Vit, West Coast Pharmaceutical Works Ltd., India (25 mg of GSH was mixed with 3 drops of SDF)	As PMI 1- SDF + GSH: was applied and agitated for 1 min, left for 2 min 2- Rinsing (water, for 30 s)				
9	Vennela [59], 2021, India	Primary teeth (*n* = 40)/carious (ICDAS scores: 5 and 6)	Storage: PBS solution	38% SDF (*n* = 10)	SDF: FAgamin, SRL, Argentina	As PMI SDF: applied for 1 min	GIC	Self-curing	Aging: Dry storage at room temperature	Outcome: - Mean gray values Timepoints: 1- Baseline (after SDF ± KI application) 2- Day 1 (after restoration) 3- After 2 w Method: Images were evaluated by ImageJ software (NIH Image, Bethesda, MD, United States of America)
				38% SDF + KI (*n* = 10)	SDF: FAgamin, SRL, Argentina KI: RIVASTAR, SDI, Bay waters, Australia	As PMI 1- SDF: applied for 1 min 2- KI: applied until the precipitate was removed 3- Washing (water)	RBC	Light-cured		
10	Nguyen [61], 2017, USA	Human molars (*n* = 27)/minimal decay	Storage: 3% bleach solution for 12 months Preparation: - Preparing class I cavities (0.5 mm into dentine)	38% SDF + KI (*n* = 4)	SDF: Advantage Arrest™, Elevate Oral Care, LLC, West Palm Beach, FL Saturated solution of KI (Lugol’s solution): 1gr/mL water or 10% by weight (Upsher-Smith, Maple Grove, MN)	1- SDF 2- KI: reapplied until no more white precipitation formed 3- Washing (water)	GIC	Self-cure GI: Riva Self Cure GI, SDI, Australia		Outcomes: 1- ∆E2000 2-∆L*/L* Timepoints: 1- Immediately after restoration 2- After 4w Only for visual examinations: 3- After 1 w 4- After 2 w 5- After 3 w Methods: 1-Visual examination using photographs 2- Color measurement: using a Nix™ Pro color sensor (Hamilton, Ontario, Canada) using industry standard 45/0° measurement
							RMGI	High-viscosity, light-cured RMGI: Riva Light Cure HV®, SDI, Australia (shade: A2)		
							RBC	- High-viscosity, light-cured RMGI: Riva Light Cure HV®, SDI, Australia - Nano-hybrid composite resin: Beautifil®, Shofu, Japan (shade of the materials: A2)	Process: 1- RMGI: 1mm, LCT: 20 s 2- Bonding system (7th generation, LCT: 10 s) 3- Filling: using incremental technique, LCT: 20 s	
				38% SDF (*n* = 4)	SDF: Advantage Arrest™, Elevate Oral Care, LLC, West Palm Beach, FL	SDF: applied to the preparation				
				Control (*n* = 1)	NA	No treatment				
11	Miller [57], 2016, USA	NM (*n* = 20)/carious (frank cavitated carious lesions)	Preparation: - Autoclaving - Removing superficial soft carious dentine	38% SDF (*n* = 10)	SDF: Riva Star Step 1, SDI Limited, Victoria, Australia	As PMI	GIC	GIC: Riva Self Cure HV, SDI Limited, Victoria, Australia	Aging: 1- TC (500 cycles) 2- Storage in artificial saliva for 30 d at 37°C	Outcome: - Intensity of staining (from 0 (no staining) to 5 (severe staining)) Timepoint: - After aging Method: Visual examination performed by three dental students based on a 6-point scale
				38% SDF + KI (*n* = 10)	SDF: Riva Star Step 1, SDI Limited, Victoria, Australia Saturated KI solution: Riva Star Step 2	1- SDF: As PMI 2- KI				

*d* day(s), *GIC* glass ionomer cement, *GSH* glutathione, *h* hour(s), *ICDAS* International Caries Detection and Assessment Score, *KI* potassium iodide, *LCT* light-curing/polymerization time, *min* minute(s), *mm* millimetre, *NA* not applicable, *NaOCl* sodium hypochlorite, *NM* not mentioned, *PBS* phosphate buffer saline, *PMI* per the manufacturer’s instructions, *RBC* resin-based composite, *RMGI(C)* resin-modified glass ionomer (cement), *s* second(s), *SDF* silver diamine fluoride, *SPM* spectrophotometer, *T(M)C* thermo(mechanical) cycling, *USA* The United States of America, *w*: week(s), *ZRGI* zirconia reinforced glass ionomer, ∆ECIEL*a*b*: ∆E calculated by CIEL*a*b* formula (∆E = [(∆L)^2^ + (∆a)^2^ + (∆b)^2^]^1/2^); ΔE2000: ∆E calculated by CIEDE2000 formula (ΔE´ = [(ΔL´/KLSL)^2^ + (ΔC´/KCSC)^2^ + (ΔH´/KHSH)^2^ + RT(ΔC´/KCSC)(ΔH´/KHSH)]^1/2^)

^a^The first author had two affiliations from different countries, we consider the first affiliation

^b^This study was not included in Table 3 due to reporting insufficient required data

Almost all studies applied 38% SDF, and only one study used both 12% and 38% SDF concentrations [55]. A total of four SDF commercial products were evaluated, with Riva Star (SDI) being most common [7, 55–57, 60]. SDF application protocols were different among studies. The reported SDF application times ranged from 1 to 3 min. [7, 21, 54–56, 59] The majority evaluated the effects of KI application following SDF (SDF + KI) [7, 21, 55, 57–61], and only one study reported the results of using a mixture of GSH and SDF [21]. Two studies compared the outcomes of delayed (up to two weeks) vs immediate restoration placement for SDF-treated samples [28, 54]. The samples in remaining studies were considered to be restored immediately, either it was directly stated or was simply mentioned that restorations were placed in the next step (without specifying the exact time).

Overall, four tooth-colored restorative materials were evaluated in the included studies (in descending order): glass ionomer cement (GIC) [21, 28, 54, 56–61], resin-based composite (RBC) [7, 28, 54–56, 59, 61], resin-modified glass ionomer (RMGI) [28, 55, 60, 61], and zirconia-reinforced glass ionomer (ZRGI) [60], with one to three materials being assessed in each report. Nine studies used light-cured dental materials during restoration in at least one of their intervention arms [7, 28, 54–56, 58–61]. In these, five reported the settings (600 mw/cm^2^ [58], 800 mw/cm^2^ [54], 1100 mw/cm^2^ and 500 nm [56]) and/or models/types (FLASHlite 1401 [55], Halogen curing light (3 M ESPE) [58], Radii-cal LED curing light [61]) of light curing units used. The most usual restoration thickness was 2 mm in four studies [28, 54, 55, 60]. Two studies stated that the (initial) class I preparations were either 0.5 mm [61] or 1 mm [58] into dentine. In three studies, cavitated carious lesions were restored [21, 57, 59]. As the cavity size was not standardized, it was assumed that the restoration thickness varied among samples in each of these three studies. A variety of aging procedures were used after restoration placement, categorized as suntest aging [56], thermo (-mechanical) cycling with additional storage in artificial saliva [55, 57], and wet/dry storage [7, 28, 54, 58, 59].

The use of dental/non-dental spectrophotometer was the predominant method of assessing color/color change in the studies, with three or four readings [7, 21, 28, 54–56, 60]. This was followed, in descending order, by visual examination [21, 57, 61], colorimeter [58, 61], and employing ImageJ software (NIH Image, Bethesda, MD, United States of America) [59]. Color change was mostly quantified by calculating ∆E using either CIEL*a*b* (ΔE﻿CIEL*a*b*) [21, 28, 56, 60] or CIEDE2000 (∆E2000) [7, 54, 55, 58, 61] formula. In these studies, ∆E values mainly presented the color change of samples in a single (sub)group at two time points [7, 28, 54–56, 60]. In one report, however, ∆Es were differences between subgroups of a single group at one assessment time [61]. Five studies also reported the values for at least one of the CIEL*a*b* color coordinates (L*, a* and b*) [28, 55, 56, 58, 61]. The first color assessments were recorded before applying any intervention materials [54, 56, 60], after SDF application [59], after restoration [7, 21, 28, 55, 58, 61], or after aging [57]. Sixty days were the maximum evaluation time in the included studies [7]. In addition, marginal discoloration [21, 61] and color difference thresholds [7, 28] were each addressed in two different reports.

### Risk of bias in studies

Table 2 presents the results of risk of bias assessment. Overall, none of the included studies judged as “sufficiently reported/adequate” for all the items in RoBDEMAT tool. It can be said that studies were moderate to low quality in general. The most frequent judgment was “insufficiently reported”. Whether the test operator was adequately blinded and the existence of negative controls were missed in the majority of studies assessed. The sample randomization, standardization of samples and materials, and identical experimental conditions across groups were significant weaknesses of the evaluated studies.

**Table 2:** Risk of bias assessment of included studies based on RoBDEMAT tool

No	Author, year of publication	D1: Bias in planning and allocation			D2: Bias in sample/specimen preparation		D3: Bias in outcome assessment		D4: Bias in data treatment and outcome reporting	
		**Control group**	**Randomization of samples**	**Sample size rationale and reporting**	**Standardization of samples and materials**	**Identical experimental conditions across groups**	**Adequate and standardized testing procedures and outcomes**	**Blinding of the test operator**	**Statistical analysis**	**Reporting study outcomes**
1	Aldosari [28], 2022	S	I	N	I	I	S	N	S	S
2	Alsagob [54], 2022	N	N	N	I	I	S	N	N	S
3	Ferreira [55], 2022	S	N	S	I	I	I	N	S	I
4	Raafat [60], 2022	N	I	S	I	I	I	NA	S	I
5	Sakr [58], 2022	N	N	N	I	I	I	N	I	I
6	Fröhlich [7], 2021	S	I	I	I	I	I	S	I	I
7	Hamdy [56], 2021	N	S	S	S	I	S	NA	S	I
8	Kamble [21], 2021	N	I	S	I	I	I	N	S	I
9	Vennela [59], 2021	N	N	N	I	I	I	N	N	I
10	Nguyen [61], 2017	S	N	N	I	I	I	N	N	I
11	Miller [57], 2016	N	I	S	I	I	I	S	S	I

*S* sufficiently reported/adequate, *I* insufficiently reported, *N* not reported/not adequate, *NA* not applicable

### Results of individual studies and data pooling

According to the reported ∆E values for 38% SDF-treated samples, in one study GIC restorations showed the lowest color change (ΔE﻿CIEL*a*b*) followed by RMGI and RBC (*P* < 0.001) [28]. In another report, ∆E2000 values measured for RBC samples were significantly less than that of GIC [54]. In one study, ZRGI restorations had significantly lower ΔE﻿CIEL*a*b* values than both GIC and RMGI (*P* < 0.001) [60]. When KI was applied following 38% SDF, significantly higher ∆E2000 values were recorded for RMGI samples compared with RBC (*P* < 0.05) [55]. Moreover, three studies showed statistically lower ∆E values at final evaluations for 38% SDF + KI- vs 38% SDF-treated samples with either RBC (*P* < 0.001) or GIC (*P* = 0.002 and *P* < 0.001) restorations [7, 21, 60]. On the contrary, the ΔE﻿CIEL*a*b* values of ZRGI and RMGI samples were statistically higher after treatment with 38% SDF + KI than when 38% SDF was used (*P* < 0.001) [60]. Delaying in restoration placement for one week, compared with their immediate application, in 38% SDF-treated samples resulted in lower ΔE﻿CIEL*a*b* values for all RBC (*P* = 0.035), RMGI (*P* = 0.201) and GIC (*P* = 0.642) restorations in one report [28]. When the two-week interval was evaluated, another study showed significantly lower ∆E2000 values for both RBC and GIC delayed restorations [54]. Additional data on the primary and secondary outcomes reported by the included studies are presented in Table 3.

**Table 3:** Summary of primary and secondary outcomes reported in the included studies

No	Author, year of publication	Tooth type (#*n* total/per (sub)group)/ caries status of dentine	Interventions	Main findings				
				**Color change**	**Color coordinates**			**Visual examination**
					Lightness (L*/∆L*)	Red-green component (a*/∆a*)	Blue-yellow component (b*/∆b*)	
1	Aldosari [28], 2022	Premolars (*n* = 90/10)/demineralized (except for control sub-groups)	Dentine pre-treatments: - Control/None - 38% SDF - 38% SDF (delayed restoration, 1 week) Restorative materials: - RBC - RMGI - GIC	∆ECIEL*a*b*: 1 day vs 7 days after restoration - Overall color stability of restorative materials: GIC > RMGI > RBC (S) - Comparing subgroups of each restorative material: 1- RBC: S 2- RMGI: S 3- GIC: NS - Delayed vs immediate restorations of SDF-treated teeth (for each restorative material): immediate > delayed: 1- RBC: S 2- RMGI: NS 3- GI: NS	L*: 1 day vs 7 days after restoration - ↓/became darker (mostly S) - GIC subgroups showed the highest lightness values	a*: 1 day vs 7 days after restoration - All values were negative/inclination to green - ↓/increase in green: RMGI (S) and GI (S/NS) subgroups - ↑/decrease in green: RBC subgroups (S/NS)	b*: 1 day vs 7 days after restoration - GIC subgroups: ↑/increase in yellow (NS) - RMGI subgroups: ↓ 1- Immediate and delayed restorations: shift to blue (S) 2- Controls: decrease in yellow (NS) - RBC: NS	-
2	Alsagob [54], 2022	Posterior permanent teeth (*n* = 40/10)/natural caries	Dentine pre-treatments: - 38% SDF - 38% SDF (delayed restoration, 2 weeks) Restorative materials: - RBC - GIC	∆E2000: before interventions vs after SDF application/after restoration/after 2 weeks/after 4 weeks - Delayed vs immediate restorations for each restorative material (after 4 weeks): immediate > delayed (S) - Comparing restorative materials with the same restoration application times (after 4 weeks): 1- Immediate: SDF + GIC > SDF + RBC (S) 2- Delayed: SDF + RBC > SDF + GIC (NS)	-	-	-	-
3	Ferreira [55], 2022	Bovine incisor teeth (*n* = 60/10)/demineralized	Dentine pre-treatments: - 12% SDF + 10% KI - 38% SDF + KI - Control/None Restorative materials: - RBC - RMGI	∆E2000: after restoration vs after TMC/30 days after TMC - Mostly↑ (S/NS) - The highest color difference comparing subgroups of each restorative material: 1- RBC: control group (for both times) 2- RMGI: 38%SDF + KI + RMGI (S, after TMC), and control group (NS, 30 days after TMC) - Comparing restorative materials with the same dentine pre-treatments: RMGI > RBC for both times (S for all comparisons of 30 days after TMC) - Comparing SDF concentrations (for each restorative material): 1- RBC: NS (for both times) 2- RMGI: S after TMC, NS 30 days after TMC	∆L*: after restoration vs after TMC/30 days after TMC - All values were negative/darkening of samples -30 days after TMC: the amounts recorded for RMGI subgroups > RBC subgroups - The smallest and largest amounts were, respectively: 1- After TMC: 38%SDF + KI + RBC and 38%SDF + KI + RMGI 2- 30 days after TMC: 12%SDF + KI + RBC and RMGI control	∆a*: after restoration vs after TMC/30 days after TMC - ↑/decrease in red: RBC and RMGI controls - Stable: other groups - The smallest and largest amounts were, respectively: 1- After TMC: RBC control/38%SDF + KI + RBC and 38%SDF + KI + RMGI 2- 30 days after TMC: RMGI control/38%SDF + KI + RBC and 38%SDF + KI + RMGI	∆b*: after restoration vs after TMC/30 days after TMC - Mostly ↑/increase in yellow - The smallest and largest amounts were, respectively: 1- After TMC: 38%SDF + KI + RBC and 12%SDF + KI + RMGI 2- 30 days after TMC: 12%SDF + KI + RMGI/38%SDF + KI + RBC and RMGI control	-
4	Raafat [60], 2022	Human premolar teeth (*n* = 36/6)/sound	Dentine pre-treatments: - 38% SDF - 38% SDF + KI Restorative material: - ZRGI - RMGI - GIC	ΔECIEL*a*b*: BL (sound dentine) vs after restoration - Comparing dentine pre-treatments for each restorative material: 1- GIC: SDF > SDF + KI (S) 2- RMGI/ZRGI: SDF + KI > SDF (S) - Comparing restorative materials with the same dentine pre-treatments: 1- SDF: RMGI > GIC > ZRGI (S) 2- SDF + KI: RMGI > ZRGI > GIC (S) - The lowest and highest values: SDF + ZRGI and SDF + KI + RMGI, respectively	-	-	-	-
5	Fröhlich [7], 2021	Bovine incisors (*n* = 15/5)/ demineralized	Dentine pre-treatments: - Control/None - 38% SDF - 38% SDF + KI Restorative material: - RBC	∆E2000: after restoration vs after 7/14/30/60 days - Comparing each dentine pre-treatment/group over time: 1- SDF + KI + RBC: NS (↑) 2- RBC/control: NS 3- SDF + RBC: NS (≥ 30 days)/S (after 60 days) - Comparing different dentine pre-treatments: 1- ≥ 30 days: NS 2- After 60 days: 2–1- SDF + RBC > SDF + KI + RBC (S) and control (S) 2–2- SDF + KI + RBC > control (NS) - Unacceptable color changes (ΔE > 1.8): after 7 days and 30 days in SDF + RBC and SDF + KI + RBC groups, respectively	-	-	-	-
6	Hamdy [56], 2021	Primary molars (*n* = 26/13)/natural caries^a^	Dentine pre-treatment: - 38% SDF Restorative materials: - RBC - GIC	ΔECIEL*a*b*: 1) BL (before interventions) vs directly after interventions, 2) directly after interventions vs after aging - Directly after intervention: both materials effectively masked the SDF-related color change - After aging: RBC performed better and its masking effect was least affected	ΔL*: 1) BL vs directly after interventions, 2) directly after interventions vs after aging - Comparisons between groups: 1- ΔL* 1: NS 2- ΔL* 2: S - Changes of L* over time (for each group): 1- Directly after interventions: ↑/ became lighter 2- After aging: ↓/ became darker (SDF + RBC showed higher value (was lighter) than its BL) 3- At both times: SDF + RBC > SDF + GIC	Δa*: 1) BL vs directly after interventions, 2) directly after interventions vs after aging - Comparisons between groups: Δa* 1/Δa* 2: S - Changes of a* over time (for each group): 1- Directly after interventions: ↓/ decrease in red for SDF + GIC and shift to green for SDF + RBC 2- After aging: all values were positive/red chroma (both groups showed values less than their BL (i.e., overall decrease in red))	Δb*: 1) BL vs directly after interventions, 2) directly after interventions vs after aging - Comparisons between groups: Δb* 1/Δb* 2: S - Changes of b* over time: 1- All values were positive/yellow chroma 2- Directly after interventions: SDF + GIC > SDF + RBC 3- After aging: SDF + RBC > SDF + GIC (SDF + RBC showed higher value than its BL (i.e., overall increase in yellow))	-
7	Kamble [21], 2021	Primary molars (*n* = 30/10)/natural caries^a^	Dentine pre-treatments: - 38% SDF - 38% SDF + KI - 38% SDF + GSH Restorative material: - GIC	ΔECIEL*a*b*: NM - Comparing each dentine pre-treatment/group over time: 1- SDF + GIC: NS (↑) 2- SDF + KI + GIC: S (↓) 3- SDF + GSH + GIC: NS - Comparisons between groups: 1- Day 1: 1–1- SDF + GIC > SDF + KI + GIC (NS) and SDF + GSH + GIC (S) 1–2- SDF + KI + GIC≈SDF + GSH + GIC 2- 1 week/4 weeks: 2–1- SDF + GIC > SDF + KI + GIC and SDF + GSH + GIC (both S) 2–2- SDF + GSH + GIC > SDF + KI + GIC (NS)	-	-	-	Assessment times: day 1, 1 week, and 4 weeks - SDF + GIC: intensification of discoloration over time - SDF + KI + GIC: no discoloration - SDF + GSH + GIC: only marginal discoloration
8	Vennela [59], 2021	Primary teeth (*n* = 40/10)/natural caries	Dentine pre-treatments: - 38%SDF - 38%SDF + KI Restorative materials: - GIC - RBC	Mean gray values: immediately after SDF ± KI application, day 1 (after restoration), and after 2 weeks - Changes in restorations over time for each group: 1- SDF + GIC/SDF + RBC: ↓/increase in black (S) 2- SDF + KI + GIC: ↓/increase in black (NS) 3- SDF + KI + RBC: NS - Comparing dentine pre-treatments for each restorative material at day 1 and day 14: 1- SDF + KI + GI > SDF + GI (S) 2- SDF + KI + RBC > SDF + RBC (NS: day 1; S: day14) - Comparing restorative materials with similar dentine pre-treatments at day 14: 1- SDF + RBC > SDF + GIC (NS) 2- SDF + KI + RBC > SDF + KI + GIC (NS) - The highest and lowest values: SDF + KI + RBC and SDF + GIC, respectively (for both times)	-	-	-	-
9	Nguyen [61], 2017	Human molars (*n* = 27/4 (interventions),1(controls))/NM	Dentine pre-treatments: - 38% SDF + KI - 38% SDF - Control/None Restorative materials: - GIC - RMGI - RBC	∆E2000 for each restorative material: SDF + KI group vs SDF group/control group after 4 weeks - Comparing SDF + KI and control groups for each restorative material: RMGI > GIC > RBC - Comparing SDF + KI and SDF groups for each restorative material: RBC > RMGI > GIC - The smallest values for each restorative material: 1- GIC: 38%SDF + KI vs 38%SDF 2- RBC/RMGI: 38%SDF + KI vs control - Overall, the smallest and largest values were related to RBC restorations (SDF + KI vs control, and SDF + KI vs SDF, respectively)	L*: immediately after restoration and after 4 weeks - ↓/became darker: SDF + GIC/RBC/RMGI - Comparing dentine pre-treatments for each restorative material (after 4 weeks): SDF + KI (lighter) > SDF	-	-	Assessment times: immediately after restoration and weekly up to 4 weeks - SDF + RBC: significant staining after light-curing (a grayish color) without any additional darkening 4 weeks later - SDF + RMGI: visible discoloration - SDF + GIC: visible marginal staining after several hours - SDF + KI + GIC/RBC/RMGI: minimal to no staining over 4 weeks - Controls: no change over time
10	Miller [57], 2016	NM (*n* = 20/10)/natural caries^a^	Dentine pre-treatments: - 38% SDF - 38% SDF + KI Restorative material: - GIC	-	-	-	-	Intensity of stainingb: after TC + storage - Comparisons between groups: NS (for both ratings of each examiner and aggregated ratings) - Mean staining intensity score: 2.5 for both groups

*BL* baseline, *GIC* glass ionomer cement, *GSH* glutathione, *KI* potassium iodide, *min* minute(s), *NM* not mentioned, *NS* statistically not significant, *RBC* resin-based composite, *RMGI* resin-modified glass ionomer, *S* statistically significant, *SDF* silver diamine fluoride, *T(M)C*: thermo(mechanical) cycling, *ZRGI* zirconia reinforced glass ionomer, ∆ECIEL*a*b*: ∆E calculated by CIEL*a*b* formula (∆E = [(∆L)^2^ + (∆a)^2^ + (∆b)^2^]^1/2^); ΔE2000: ∆E calculated by CIEDE2000 formula (ΔE´ = [(ΔL´/KLSL)2 + (ΔC´/KCSC)2 + (ΔH´/KHSH)2 + RT(ΔC´/KCSC)(ΔH´/KHSH)]1/2)

^a^Some preparations were performed, ^b^. 0  = no staining, 5 = severe staining

**Fig. 2:**
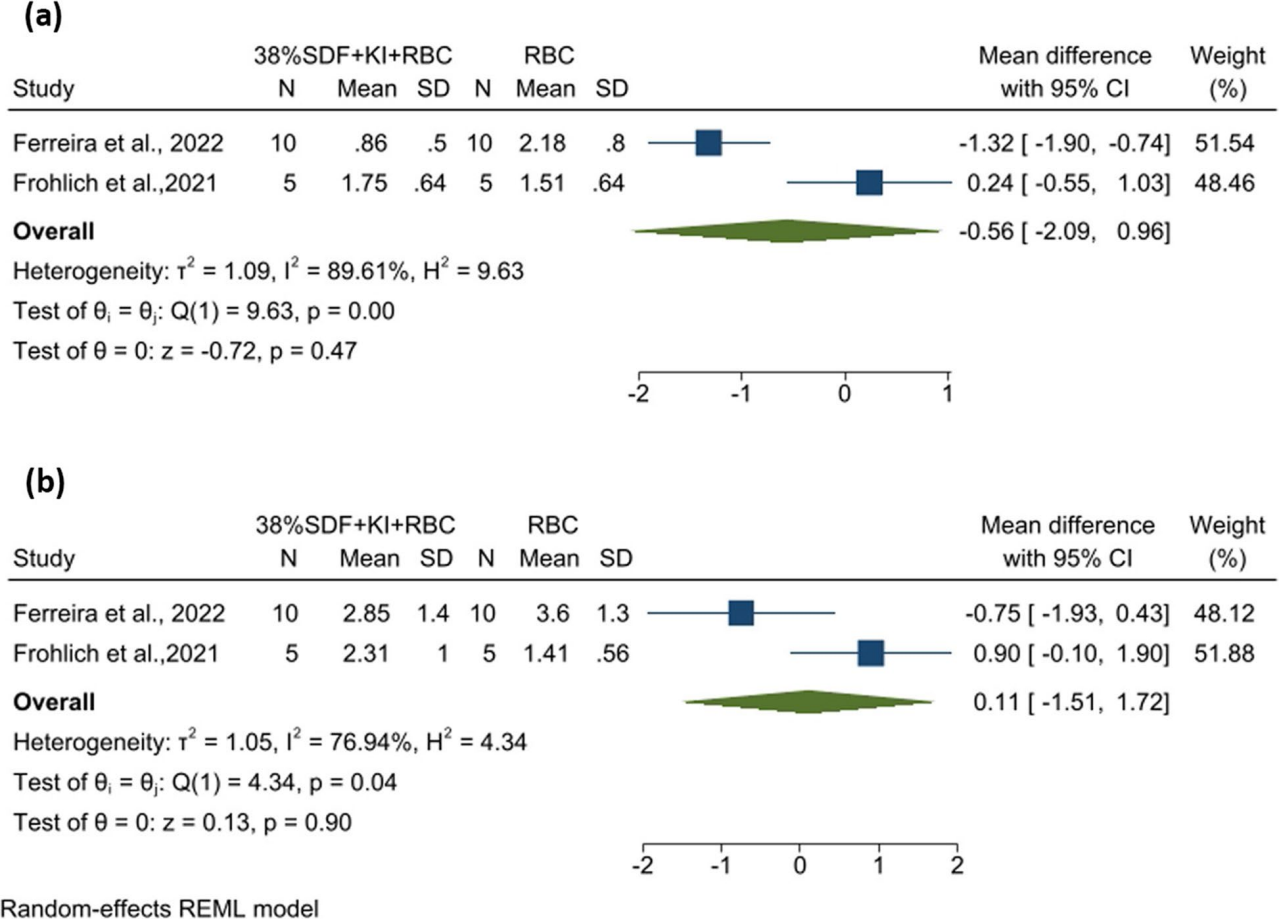
Data pooling for comparisons between resin-based composite (RBC) restorations with and without prior 38% silver diamine fluoride and potassium iodide (38% SDF + KI) application (**a**) at ~ 14 days, and (**b**) at ~ 60 days. CI: confidence interval; *N*: the number of samples in each group; SD: standard deviation

Data pooling was performed for comparisons evaluating RBC restorations with and without prior 38% SDF + KI application at two various time points (Fig. 2) [7, 55]. The outcome measure was color difference presented as ΔE2000 (differences between color assessments after restoration vs ~ 14 days/ ~ 60 days after restoration). For the study by Ferreira et al. [55], the duration of thermomechanical cycling was estimated to be 18 days, therefore the second and third assessment times were ~ 19 and ~ 49 days following restoration. Overall, the analyses showed no statistically significant differences between RBC and 38%SDF + KI + RBC samples at both time points (~ 14 days: WMD: -0.56, 95% CI: -2.09 to 0.96, *P* = 0.47; ~ 60 days: WMD: 0.11, 95% CI: -1.51 to 1.72, *P* = 0.90). The heterogeneity in both analyses was high (~ 14 days: I^2^: 89.6%, *P* = 0.002; ~ 60 days: I^2^: 76.9%, *P* = 0.037). In addition, the results indicated that the pooled ∆E of 38% SDF + KI + RBC group was less than that of RBC at ~ 14 days. This association reversed at ~ 60 days, that is, the pooled ∆E of 38% SDF + KI + RBC group turned greater (Fig. 2).

## Discussion

The results of included studies in our systematic review on color and/or color change of tooth-colored restorations post application of SDF on dentine were inconclusive overall. This is explained by variations in study settings, possibly affecting the amount of SDF-related dentine discoloration produced, relevant optical properties of restorations, and comparability of outcomes. The pooled data for comparisons of 38% SDF + KI + RBC vs RBC restorations revealed no statistically significant differences in color changes at short- and long-term intervals. The results further indicated an increase in SDF-related discoloration over time. These findings can be explained by the ability of KI in reversing SDF color change [7, 19, 21, 59]. KI is expected to prevent the formation of silver oxide and subsequently reduce black staining through reacting with excess silver ions and precipitating yellow silver iodide [34]. However, this effect may be influenced by the amount of KI applied and is temporary possibly due to the high photosensitivity of produced silver iodide [55, 62].

Various protocols were described for storage and/or disinfection/sterilization of collected extracted teeth, with several of solutions/methods possibly reacting with SDF or altering its interaction with dentine. Four studies used solutions containing Cl ions (namely, phosphate buffer saline, sodium hypochlorite and chloramine T) for storage [28, 59, 61] or only for disinfecting the teeth [21]. Silver ions from SDF solution applied on the tooth surface can react with Cl ions retained in the tooth structure following storage/disinfection process and form silver chloride which can reduce the SDF-related black staining [16, 35, 59]. The amount of Cl ions available for reacting with silver was expected to vary among these studies due to differences in storage times and solutions. In addition, one study autoclaved the carious teeth collected [57]. It was shown that autoclaving can lead to denaturation of dentine collagen in sound teeth with no effects on dentine permeability. In cases where dentine collagen was exposed, as in carious teeth, autoclaving caused the denatured collagen network/mesh to become compact and collapsed with consequent reduction in dentine permeability [63]. This probably alters SDF penetration [57], however, its effects on the amount of final SDF discoloration should be investigated.

Most included studies used carious or demineralized dentine substrates [7, 21, 28, 54–57, 59]. Only one eligible study used carious teeth with no further mechanical preparations [59], while others sectioned [54], performed complete (for cavity walls) or partial caries removal [21, 57], or prepared carious dentine slices [56]. In addition, demineralized dentine specimens were prepared from sound teeth through submitting to either demineralization or demineralization/remineralization regimen for 7–14 days [7, 28, 55]. These variations in methodology may produce significant differences in the amount/degree of demineralization in dentine substrates across the studies [64]. The degree of dentine demineralization affects SDF-related color change [36]. The higher the degree of demineralization the greater the amount of color change and discoloration depth; this can be explained by the high affinity of silver ions for collagen/proteins and greater amount of exposed collagen available [36, 65].

Dentine treatments prior to restoration placement were also different among included reports. Only four studies incorporated negative controls [7, 28, 55, 61]. In general, studies with no control groups have limited value [32]. In most reports, dental/non-dental KI products were applied following SDF at least in one intervention group [7, 21, 55, 57–61]. The KI concentration should be considered when interpreting the results of studies. Although the differences between KI concentrations in reducing SDF black staining might become less pronounced over time, saturated KI solution can still show significant differences compared with lower concentrations [16]. The concentrations of the products used with 38% SDF (namely, Riva Star step 2 and saturated KI/Lugol’s solution) in the included studies were similar [34]. A mixture of GSH and SDF was assessed in one study [21]. GSH is a most common intracellular non-protein thiols acting as a reducing agent in mammalian cells [22]. This mixture of GSH and SDF, compared with SDF alone, statistically reduced SDF-related dentine black discoloration with/without tooth-colored restorations [21, 22]. It is speculated that GSH decrease SDF discoloration through reducing silver aggregation and controlling its release [22]. However, its inability to completely overcome SDF-related discoloration have been attributed to the insufficient amount of GSH mixed with SDF [21, 22].

SDF application methods varied among the eligible reports. These variations might be the results of differences in available protocols which are principally based on expert’s opinions [66]. Overall, the 1- to 3-min application times used in the included studies were in accordance with most available recommendations for SDF therapy to caries arrest [66]. In two studies, SDF-treated samples were water-rinsed for 30 s [21, 56]. Although rinsing/washing following SDF application is not widely advocated [66], it might be considered when restoring SDF-treated lesions with RBCs [67]. In one study SDF solution was first agitated and then left for additional time on tooth surface [21]. The rationale for scrubbing SDF during its application is to release/reduce surface tension [11] and therefore increasing the wettability of solution. However, the impacts of such variations on SDF dentine discoloration should also be reported in future studies, as merely reporting silver penetration depth does not resemble depth and amount of discoloration at least in short observation times [36, 65].

Four types and a variety of commercial products of tooth-colored restorative materials were evaluated in the included studies. In almost all the studies, one single restorative material/product was applied in each group. The translucency, masking ability, and color stability are properties affecting the final color of restorations bonded to SDF-treated dentine. Conventional GICs are opaque materials, while RMGIs are more translucent. In addition, RBCs are now supplied with a variety of translucencies/opacities [33]. Therefore, translucency/opacity of restorative materials (or probably restorations) will be different across the board. Masking ability is influenced by material thickness [33]. The reported restoration thickness was 1 to 2 mm in five studies [7, 28, 54, 55, 60], and 4 mm in one report [56]. The other three studies restored non-standardized cavitated carious teeth with non-recorded depths [21, 57, 59]. On a clinical level, in anterior teeth, especially in primary dentition, there is limited space for restorations of SDF-treated teeth, therefore, considering both material thickness and translucency is critical to achieve acceptable esthetic outcomes [33, 68]. A recent in vitro study showed that pink opaquer with or without RBC at 2 mm thickness produced clinically acceptable results for masking the simulated SDF discoloration. However, the results for RMGI and opaque-shade RBC were not promising in that study; therefore, their use was not recommended [33]. The masking ability of RBCs is also affected by layering strategy and substrate color. The layering technique is commonly used to promote natural lifelike restorations, and thicker layers of opaque shades enhance the masking ability of final restorations [69]. However, none of the included studies applied the layering technique. Only in one study, a layer of RMGI was placed prior to restoring cavities with an RBC material [61]. Due to their translucency, the masking of underlying dark substrates, as in SDF-arrested/treated dentine, is challenging when RBCs are used. Increasing the thickness of the dentine shade layer or the combined application of RBC and opaquers is recommended to achieve acceptable results [69]. Color stability is affected by internal (mainly material composition) and/or external (including environment and material manipulation) factors [27, 70]. Due to its composition, RBC is considered to be more color stable than GIC [55]. In the eligible studies, all reported media used for storage of restored samples were colorless solutions (either water or artificial saliva). In three studies dry samples were used for color assessments [56, 59, 60]. In fact, these studies recorded absolute staining potential of SDF, which might be slightly different from the results of non-dry sample assessments [56].

Light curing of materials used during restoration following SDF application caused immediate grayish discoloration [56, 61], which is noteworthy. This finding is attributed to the photosensitivity of SDF and the accelerated production of black metallic silver by light exposure [56]. To avoid the latter staining, delaying tooth restoration has been proposed [11]. In this review, RBC restorations placed one to two weeks after SDF applications had significantly less color differences than those placed immediately [28, 54].

Instrumental color assessment, acquired by a spectrophotometer (most common), a colorimeter, or photographs/ImagJ software, was performed in almost all the included studies. Spectrophotometers are one of the most accurate instruments for dental/non-dental color matching, measuring the reflected light in the visible spectrum [71]. Colorimeters register only three colors from the visible spectrum, and therefore, are generally less accurate than spectrophotometers [71, 72]. Two studies employed both visual and instrumental color assessments [21, 61]. It is recommended to use both assessment methods, if possible, for color matching as they complement each other [71]. Several included studies used photographs in their color assessment procedures [21, 58, 59, 61]. Using photographic images for color assessment/analysis, one innate difficulty might be capturing images with identical environmental lighting parameters. Otherwise, deviations in the results would generally be expected [17]. The studies included in this systematic review commonly described photography equipment they used; however, they did not report photography conditions. 

∆E, the most reported outcome, was calculated with either CIEL*a*b* or CIEDE2000 formula. CIEDE2000 formula is the latest color difference formula developed to overcome the weakness of the L*a*b* color space, that is, discrepancies existing between the measurement results and visual examinations [27]. In this review, one more study used CIEDE2000 formula compared with those using the older CIEL*a*b* formula. ∆E values alone are of limited clinical importance. It has been recommended by ISO to interpret numerical data based on color difference thresholds [68]. Only two studies addressed this issue [7, 28]. Moreover, ∆Es do not provide sufficient information regarding the direction of color change [56]. Accordingly, several included studies also reported data on individual color parameters to provide more information in this regard [28, 55, 56, 58, 61]. Time points color measurements performed varied considerably among included studies.

The main limitation of this systematic review was the heterogeneity encountered across the included studies primarily due to variations in dentine pre-treatments and restorative materials evaluated/applied, and outcomes reported (including the diversity in the assessment times), refraining us from performing meta-analysis. Systematic reviews of in vitro studies often face this heterogeneity [67]. There were also scant clinical studies on this topic. Therefore, this review was based on laboratory data; an inherent limitation as their results cannot be directly extrapolated to clinical situations [29]. Furthermore, only color/color change of tooth-colored restorations bonded to SDF-treated dentine was evaluated in this review. These data should be considered along with the results of relevant systematic reviews on bond strength of such restorations [67, 73].

Researchers are encouraged to precisely report relevant details of their future in vitro studies, including information on blindness of outcome assessor, storage/disinfection/aging conditions, and methods of sample randomization and standardizing samples. Moreover, considering negative controls for future research is highly recommended. We can advocate that forthcoming studies may compare different tooth-colored/esthetic restorative materials and evaluate layered restorations. Simulating clinical conditions, adhering to clinically established SDF application guidelines and reporting outcomes at different time intervals, including long-term time points, will provide more valuable data that can be statistically analyzed. Finally, evaluating possible structural changes of both restorative materials and SDF-treated dentine substrates following restoration, especially in case of immediate restoration placement, over time from color change aspect in future in vitro studies will enhance our understanding of the underlying mechanisms of restoration color change.

## Conclusions

No definite conclusions can be drawn on color/color change of tooth-colored restorative materials applied following SDF application on dentine, due to substantial setting and conceptual heterogeneity across the included studies. According to the limited evidence, RBC restorations alone and following the application of 38% SDF with KI performed comparably up to 60 days. Future high-quality studies with considerable sample size and longer follow-up comparing color differences of different tooth-colored restorative materials placed after SDF application (associated with KI or other modifications) are imperative to find solutions for reducing the impact of SDF-related staining on final tooth-colored restorations, especially on anterior teeth. There is also need for clinical studies on this topic.

### Supplementary Information

Supplementary Material 1.

## Data Availability

The datasets used and/or analysed during the current study are available from the corresponding author on reasonable request.

## Abbreviations

CI: Confidence interval
GIC: Glass ionomer cement
GSH: Glutathione
KI: Potassium iodide
MID: Minimal intervention dentistry
RBC: Resin-based composite
RMGI: Resin-modified glass ionomer
SDF: Silver diamine fluoride
WMD: Weighted mean difference
ZRGI: Zirconia-reinforced glass ionomer
∆E: Color difference
∆ECIEL*a*b*: ∆E calculated by CIEL*a*b* formula
∆E2000: ∆E calculated by CIEDE2000 formula
