# Supplementary material for: Color change of tooth-colored restorative materials bonded to silver diamine fluoride-treated dentine: a systematic review
Source: BMC Oral Health. 2024 Jun 27;24:737. doi: 10.1186/s12903-024-04487-0 (PMC11210045; doi:10.1186/s12903-024-04487-0)
Supplement: Supplementary file 1 — Supplementary Material 1. [file 12903_2024_4487_MOESM1_ESM.docx]

**Supplementary material 1: Search strategy**

| **1. PubMed** | | | |
| --- | --- | --- | --- |
| **No.** | **Query** | **Search details** | **No. of retrieved records** |
| #1 | ("silver diamine fluoride" OR "silver diammine fluoride" OR "silver ammonia fluoride" OR "diamine silver fluoride" OR "diammine silver fluoride" OR "silver fluoride" OR "SDF") | "silver diamine fluoride"[All Fields] OR "silver diammine fluoride"[All Fields] OR "silver ammonia fluoride"[All Fields] OR "diamine silver fluoride"[All Fields] OR "diammine silver fluoride"[All Fields] OR "silver fluoride"[All Fields] OR "SDF"[All Fields] | 8,721 |
| #2 | (masking OR mask OR masked OR color OR colour OR discoloration OR discolored OR discolouration OR discoloured OR staining OR stained OR stain OR esthetic OR aesthetic OR visual OR restoration OR restorative OR restored OR filling OR filled) | "mask s"[All Fields] OR "masked"[All Fields] OR "masking"[All Fields] OR "masks"[MeSH Terms] OR "masks"[All Fields] OR ("masks"[MeSH Terms] OR "masks"[All Fields] OR "mask"[All Fields]) OR ("mask s"[All Fields] OR "masked"[All Fields] OR "masking"[All Fields] OR "masks"[MeSH Terms] OR "masks"[All Fields]) OR ("colorant"[All Fields] OR "colorants"[All Fields] OR "coloration"[All Fields] OR "colorations"[All Fields] OR "colored"[All Fields] OR "coloreds"[All Fields] OR "colorful"[All Fields] OR "colorfulness"[All Fields] OR "coloring"[All Fields] OR "colorings"[All Fields] OR "colorization"[All Fields] OR "colorized"[All Fields] OR "colour"[All Fields] OR "color"[MeSH Terms] OR "color"[All Fields] OR "colourant"[All Fields] OR "colourants"[All Fields] OR "colouration"[All Fields] OR "colourations"[All Fields] OR "coloured"[All Fields] OR "coloureds"[All Fields] OR "colourful"[All Fields] OR "colourfulness"[All Fields] OR "colouring"[All Fields] OR "colourings"[All Fields] OR "colours"[All Fields] OR "colors"[All Fields]) OR ("colorant"[All Fields] OR "colorants"[All Fields] OR "coloration"[All Fields] OR "colorations"[All Fields] OR "colored"[All Fields] OR "coloreds"[All Fields] OR "colorful"[All Fields] OR "colorfulness"[All Fields] OR "coloring"[All Fields] OR "colorings"[All Fields] OR "colorization"[All Fields] OR "colorized"[All Fields] OR "colour"[All Fields] OR "color"[MeSH Terms] OR "color"[All Fields] OR "colourant"[All Fields] OR "colourants"[All Fields] OR "colouration"[All Fields] OR "colourations"[All Fields] OR "coloured"[All Fields] OR "coloureds"[All Fields] OR "colourful"[All Fields] OR "colourfulness"[All Fields] OR "colouring"[All Fields] OR "colourings"[All Fields] OR "colours"[All Fields] OR "colors"[All Fields]) OR ("discolor"[All Fields] OR "discoloration"[All Fields] OR "discolorations"[All Fields] OR "discolored"[All Fields] OR "discoloring"[All Fields] OR "discolors"[All Fields] OR "discolour"[All Fields] OR "discolouration"[All Fields] OR "discolourations"[All Fields] OR "discoloured"[All Fields] OR "discolouring"[All Fields]) OR ("discolor"[All Fields] OR "discoloration"[All Fields] OR "discolorations"[All Fields] OR "discolored"[All Fields] OR "discoloring"[All Fields] OR "discolors"[All Fields] OR "discolour"[All Fields] OR "discolouration"[All Fields] OR "discolourations"[All Fields] OR "discoloured"[All Fields] OR "discolouring"[All Fields]) OR ("discolor"[All Fields] OR "discoloration"[All Fields] OR "discolorations"[All Fields] OR "discolored"[All Fields] OR "discoloring"[All Fields] OR "discolors"[All Fields] OR "discolour"[All Fields] OR "discolouration"[All Fields] OR "discolourations"[All Fields] OR "discoloured"[All Fields] OR "discolouring"[All Fields]) OR ("discolor"[All Fields] OR "discoloration"[All Fields] OR "discolorations"[All Fields] OR "discolored"[All Fields] OR "discoloring"[All Fields] OR "discolors"[All Fields] OR "discolour"[All Fields] OR "discolouration"[All Fields] OR "discolourations"[All Fields] OR "discoloured"[All Fields] OR "discolouring"[All Fields]) OR ("coloring agents"[Pharmacological Action] OR "coloring agents"[MeSH Terms] OR ("coloring"[All Fields] AND "agents"[All Fields]) OR "coloring agents"[All Fields] OR "stains"[All Fields] OR "stained"[All Fields] OR "staining and labeling"[MeSH Terms] OR ("staining"[All Fields] AND "labeling"[All Fields]) OR "staining and labeling"[All Fields] OR "stain"[All Fields] OR "staining"[All Fields] OR "stainings"[All Fields] OR "staining s"[All Fields] OR "stainning"[All Fields]) OR ("coloring agents"[Pharmacological Action] OR "coloring agents"[MeSH Terms] OR ("coloring"[All Fields] AND "agents"[All Fields]) OR "coloring agents"[All Fields] OR "stains"[All Fields] OR "stained"[All Fields] OR "staining and labeling"[MeSH Terms] OR ("staining"[All Fields] AND "labeling"[All Fields]) OR "staining and labeling"[All Fields] OR "stain"[All Fields] OR "staining"[All Fields] OR "stainings"[All Fields] OR "staining s"[All Fields] OR "stainning"[All Fields]) OR ("coloring agents"[Pharmacological Action] OR "coloring agents"[MeSH Terms] OR ("coloring"[All Fields] AND "agents"[All Fields]) OR "coloring agents"[All Fields] OR "stains"[All Fields] OR "stained"[All Fields] OR "staining and labeling"[MeSH Terms] OR ("staining"[All Fields] AND "labeling"[All Fields]) OR "staining and labeling"[All Fields] OR "stain"[All Fields] OR "staining"[All Fields] OR "stainings"[All Fields] OR "staining s"[All Fields] OR "stainning"[All Fields]) OR ("aesthetical"[All Fields] OR "aesthetically"[All Fields] OR "esthetical"[All Fields] OR "esthetically"[All Fields] OR "esthetics"[MeSH Terms] OR "esthetics"[All Fields] OR "aesthetic"[All Fields] OR "aesthetics"[All Fields] OR "esthetic"[All Fields]) OR ("aesthetical"[All Fields] OR "aesthetically"[All Fields] OR "esthetical"[All Fields] OR "esthetically"[All Fields] OR "esthetics"[MeSH Terms] OR "esthetics"[All Fields] OR "aesthetic"[All Fields] OR "aesthetics"[All Fields] OR "esthetic"[All Fields]) OR ("visual"[All Fields] OR "visualisation"[All Fields] OR "visualisations"[All Fields] OR "visualise"[All Fields] OR "visualised"[All Fields] OR "visualises"[All Fields] OR "visualising"[All Fields] OR "visualization"[All Fields] OR "visualizations"[All Fields] OR "visualize"[All Fields] OR "visualized"[All Fields] OR "visualizer"[All Fields] OR "visualizers"[All Fields] OR "visualizes"[All Fields] OR "visualizing"[All Fields] OR "visually"[All Fields] OR "visuals"[All Fields]) OR ("restorability"[All Fields] OR "restorable"[All Fields] OR "restorated"[All Fields] OR "restoration"[All Fields] OR "restoration s"[All Fields] OR "restorations"[All Fields] OR "restorative"[All Fields] OR "restoratives"[All Fields] OR "restore"[All Fields] OR "restored"[All Fields] OR "restores"[All Fields] OR "restoring"[All Fields]) OR ("restorability"[All Fields] OR "restorable"[All Fields] OR "restorated"[All Fields] OR "restoration"[All Fields] OR "restoration s"[All Fields] OR "restorations"[All Fields] OR "restorative"[All Fields] OR "restoratives"[All Fields] OR "restore"[All Fields] OR "restored"[All Fields] OR "restores"[All Fields] OR "restoring"[All Fields]) OR ("restorability"[All Fields] OR "restorable"[All Fields] OR "restorated"[All Fields] OR "restoration"[All Fields] OR "restoration s"[All Fields] OR "restorations"[All Fields] OR "restorative"[All Fields] OR "restoratives"[All Fields] OR "restore"[All Fields] OR "restored"[All Fields] OR "restores"[All Fields] OR "restoring"[All Fields]) OR ("filled"[All Fields] OR "filling"[All Fields] OR "fillings"[All Fields] OR "fills"[All Fields]) OR ("filled"[All Fields] OR "filling"[All Fields] OR "fillings"[All Fields] OR "fills"[All Fields]) | 2,655,993 |
| #3 | #1 AND #2 | | 1,467 |

| **2. Embase** | | | |
| --- | --- | --- | --- |
| **No.** | **Query** | **Search details** | **No. of retrieved records** |
| #1 | ("silver diamine fluoride" OR "silver diammine fluoride" OR "silver ammonia fluoride" OR "diamine silver fluoride" OR "diammine silver fluoride" OR "silver fluoride" OR "SDF") | 'silver diamine fluoride'/exp OR 'silver diamine fluoride' OR 'silver diammine fluoride' OR 'silver ammonia fluoride' OR 'diamine silver fluoride' OR 'diammine silver fluoride' OR 'silver fluoride'/exp OR 'silver fluoride' OR 'sdf' | 12,607 |
| #2 | (masking OR mask OR masked OR color OR colour OR discoloration OR discolored OR discolouration OR discoloured OR staining OR stained OR stain OR esthetic OR aesthetic OR visual OR restoration OR restorative OR restored OR filling OR filled) | 'masking'/exp OR masking OR 'mask'/exp OR mask OR masked OR 'color'/exp OR color OR 'colour'/exp OR colour OR 'discoloration'/exp OR discoloration OR discolored OR discolouration OR discoloured OR 'staining'/exp OR staining OR stained OR 'stain'/exp OR stain OR esthetic OR aesthetic OR visual OR 'restoration'/exp OR restoration OR restorative OR restored OR filling OR filled | 2,899,265 |
| #3 | #1 AND #2 | | 1,882 |

| **3. ISI Web of Science (all databases)** | | | |
| --- | --- | --- | --- |
| **No.** | **Query** | **Search details** | **No. of retrieved records** |
| #1 | ("silver diamine fluoride" OR "silver diammine fluoride" OR "silver ammonia fluoride" OR "diamine silver fluoride" OR "diammine silver fluoride" OR "silver fluoride" OR "SDF") | "silver diamine fluoride" OR "silver diammine fluoride" OR "silver ammonia fluoride" OR "diamine silver fluoride" OR "diammine silver fluoride" OR "silver fluoride" OR "SDF" (Topic) and Preprint Citation Index (Exclude – Database) | 19,309 |
| #2 | (masking OR mask OR masked OR color OR colour OR discoloration OR discolored OR discolouration OR discoloured OR staining OR stained OR stain OR esthetic OR aesthetic OR visual OR restoration OR restorative OR restored OR filling OR filled) | masking OR mask OR masked OR color OR colour OR discoloration OR discolored OR discolouration OR discoloured OR staining OR stained OR stain OR esthetic OR aesthetic OR visual OR restoration OR restorative OR restored OR filling OR filled (Topic) and Preprint Citation Index (Exclude – Database) | 8,087,149 |
| #3 | #1 AND #2 | | 2,756 |

| **4. Scopus** | | | |
| --- | --- | --- | --- |
| **No.** | **Query** | **Search details** | **No. of retrieved records** |
| #1 | ("silver diamine fluoride" OR "silver diammine fluoride" OR "silver ammonia fluoride" OR "diamine silver fluoride" OR "diammine silver fluoride" OR "silver fluoride" OR "SDF") | TITLE-ABS-KEY ( ( "silver diamine fluoride" OR "silver diammine fluoride" OR "silver ammonia fluoride" OR "diamine silver fluoride" OR "diammine silver fluoride" OR "silver fluoride" OR "SDF" ) ) | 12,827 |
| #2 | (masking OR mask OR masked OR color OR colour OR discoloration OR discolored OR discolouration OR discoloured OR staining OR stained OR stain OR esthetic OR aesthetic OR visual OR restoration OR restorative OR restored OR filling OR filled) | TITLE-ABS-KEY ( ( masking OR mask OR masked OR color OR colour OR discoloration OR discolored OR discolouration OR discoloured OR staining OR stained OR stain OR esthetic OR aesthetic OR visual OR restoration OR restorative OR restored OR filling OR filled ) ) | 4,389,262 |
| #3 | #1 AND #2 | | 1,408 |

**Supplementary material 2: Reasons for excluding reports after full-text evaluation**

| **No.** | **Author, publication year** | **Main reason for exclusion** |
| --- | --- | --- |
|  | Camatta,^37^ 2023 | Samples were not restored following SDF treatment |
|  | Islam,^52^ 2023 | Samples were not restored following SDF treatment |
|  | Asghar,^40^ 2022 | Samples were not restored following SDF treatment |
|  | de Almeida,^39^ 2022 | Samples were not restored following SDF treatment |
|  | Andijani,^50^ 2021 | Samples were not restored following SDF treatment |
|  | Cripps,^51^ 2021 | Simulated SDF discoloration was evaluated/no SDF solutions were applied |
|  | Detsomboonrat,^16^ 2022 | Samples were not restored following SDF treatment |
|  | Lim,^33^ 2022 | Simulated SDF discoloration was evaluated/no SDF solutions were applied |
|  | Luong,^30^ 2022 | Samples were not restored following SDF treatment |
|  | Molina,^38^ 2022 | Color assessments were performed before restoration placement |
|  | Espindola,^23^ 2020 | Samples were not restored following SDF treatment |
|  | Nizami,^45^ 2020 | Did not assessed color of SDF-treated samples |
|  | Sayed,^44^ 2020 | Samples were not restored following SDF treatment |
|  | Sayed,^43^ 2020 | Samples were not restored following SDF treatment |
|  | Yin,^42^ 2020 | Samples were not restored following SDF treatment |
|  | Zhao,^41^ 2020 | Samples were not restored following SDF treatment |
|  | Sayed,^36^ 2019 | Samples were not restored following SDF treatment |
|  | Shitomi,^47^ 2019 | Samples were not restored following SDF treatment |
|  | Zhao,^46^ 2019 | Color assessments were performed before restoration placement |
|  | McDonald,^53^ 2018 | Enamel discoloration was evaluated |
|  | Patel,^35^ 2018 | Samples were not restored following SDF treatment |
|  | Sayed,^22^ 2018 | Samples were not restored following SDF treatment |
|  | Wierichs,^48^ 2018 | Samples were not restored following SDF treatment |
|  | Zhao,^29^ 2017 | Optical properties were not evaluated (only marginal discoloration was assessed) |
|  | Kawasaki,^49^ 2005 | No color assessments were performed |

SDF: silver diammine fluoride
